# High space–time bandwidth product imaging in low coherence quantitative phase microscopy

**DOI:** 10.1038/s41598-024-59874-y

**Published:** 2024-04-22

**Authors:** Azeem Ahmad, Paweł Gocłowski, Vishesh Dubey, Maciej Trusiak, Balpreet S. Ahluwalia

**Affiliations:** 1 Department of Physics and Technology, UiT The Arctic University of Norway, 9037 Tromsø, Norway; 2 Institute of Micromechanics and Photonics, Warsaw University of Technology, 8 Sw. A. Boboli St., 02-525 Warsaw, Poland

**Keywords:** Applied optics, Lasers, LEDs and light sources, Optical techniques, Microscopy, Optics and photonics, Biophotonics

## Abstract

Current low coherence quantitative phase microscopy (LC-QPM) systems suffer from either reduced field of view (FoV) or reduced temporal resolution due to the short temporal coherence (TC) length of the light source. Here, we propose a hybrid, experimental and numerical approach to address this core problem associated with LC-QPM. We demonstrate high spatial resolution and high phase sensitivity in LC-QPM at high temporal resolution. High space–time bandwidth product is achieved by employing incoherent light source for sample illumination in QPM to increase the spatial resolution and single-shot Hilbert spiral transform (HST) based phase recovery algorithm to enhance the temporal resolution without sacrificing spatial resolution during the reconstruction steps. The high spatial phase sensitivity comes by default due to the use of incoherent light source in QPM which has low temporal coherence length and does not generate speckle noise and coherent noise. The spatial resolution achieved by the HST is slightly inferior to the temporal phase-shifting (TPS) method when tested on a specimen but surpasses that of the single-shot Fourier transform (FT) based phase recovery method. Contrary to HST method, FT method requires high density fringes for lossless phase recovery, which is difficult to achieve in LC-QPM over entire FoV. Consequently, integration of HST algorithm with LC-QPM system makes an attractive route. Here, we demonstrate scalable FoV and resolution in single-shot LC-QPM and experimentally corroborate it on a test object and on both live and fixed biological specimen such as MEF, U2OS and human red blood cells (RBCs). LC-QPM system with HST reconstruction offer high-speed single-shot QPM imaging at high phase sensitivity and high spatial resolution enabling us to study sub-cellular dynamic inside U2OS for extended duration (3 h) and observe high-speed (50 fps) dynamics of human RBCs. The experimental results validate the effectiveness of the present approach and will open new avenues in the domain of biomedical imaging in the future.

## Introduction

During the last two decades, quantitative phase microscopy (QPM), which is a label free optical microscopy technique, has attracted a strong attention of the researchers worldwide due to its non-contact, non-invasive and quantitative nature^1–3^. QPM has the capability to precisely quantify various morphological parameters (such as surface area, volume, sphericity etc.), dry mass and phase related texture parameters (such as mean, standard deviation, kurtosis etc.) of the biological specimens^1,4^. The precise label-free quantification of these parameters strongly depends on the phase sensitivity which is influenced by the type of illumination (i.e., coherent and incoherent light source) utilized in QPM^3,5–7^.

**Figure 1:**
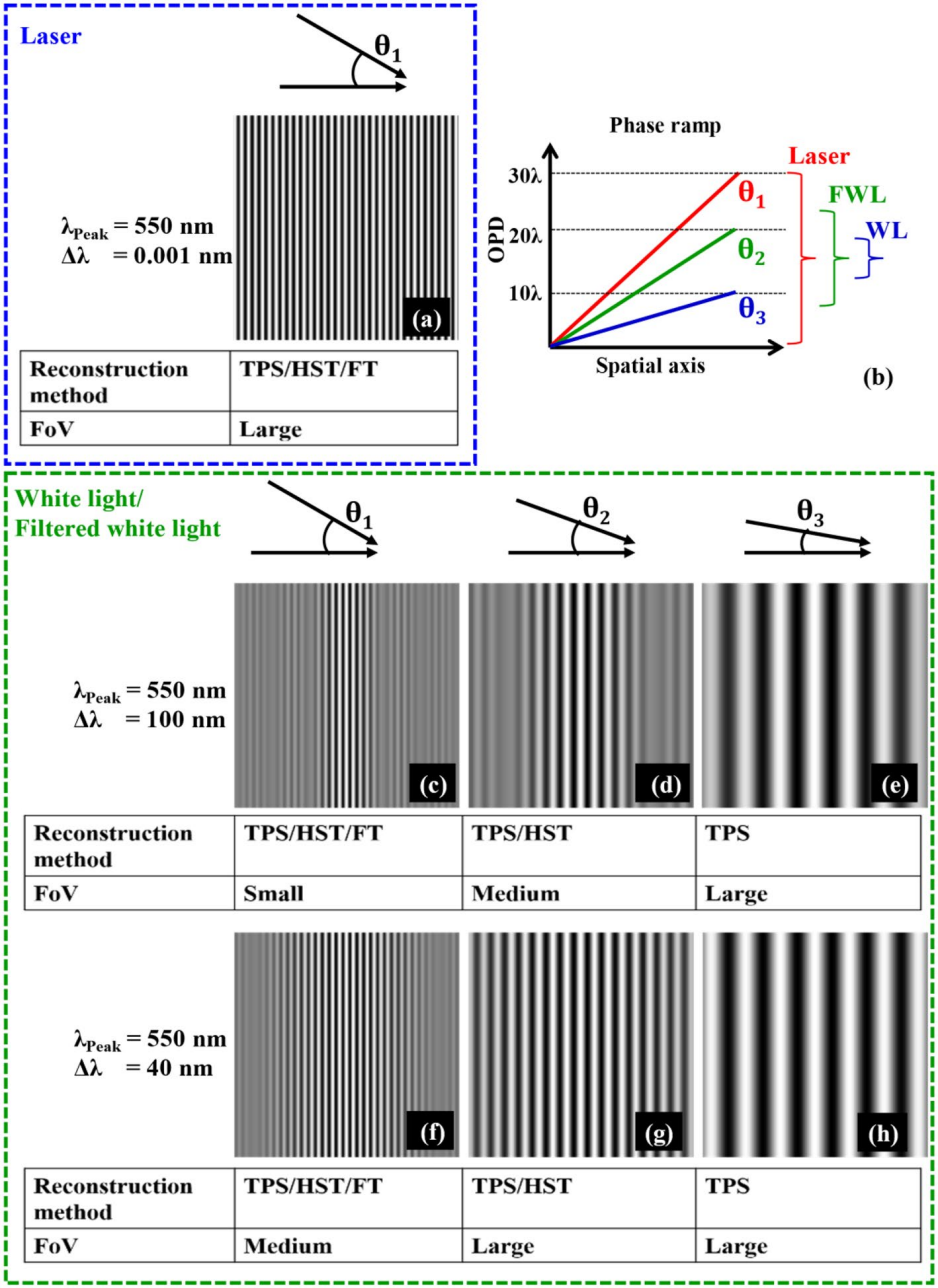
Concept Figure: high coherence versus low coherence. The simulation study for the comparison of different light sources in terms of spectral bandwidth and fringe density. It exhibits the requirement of right fringe density for the lossless phase recovery using different phase reconstruction algorithms such as TPS, HST, and FT and its influence on supported FoV.

Coherent light sources, i.e. lasers, have been extensively used in QPM systems due to their high temporal and high spatial coherence properties, which ease the formation of interference fringes in QPM^3^. In addition, high density fringes can be easily obtained to implement single-shot Fourier transform (FT) based phase recovery algorithm (Fig. 1a)^8^. However, high coherence properties of lasers generate speckle noise and spurious fringes in QPM and reduce the spatial phase sensitivity significantly^3^. On the contrary, low and incoherent light sources such as LEDs and white light (WL) lamps enable high spatial phase sensitivity in QPM^5,7,9–11^. However, the low temporal coherence (TC) property of these light sources puts a strict condition, i.e., optical path difference (OPD) between the object and the reference arm must be smaller than the TC length of the light source to form sharp interference fringes in QPM. Thus, low TC length of these light sources restricts the use of identical objective lenses in the object and the reference arm of QPM^12^. Moreover, low TC length confines the interference fringes in a small FoV if high density fringes are required - for example when using FT based phase recovery algorithm for lossless phase reconstruction. This is due to the large phase ramp required for FT fringe pattern generation; and high contrast interference fringes are obtained only within small FoV where OPD values are smaller than TC length of the light source (Fig. 1b,c,f). The area of interference FoV can be increased by reducing the phase ramp as depicted in Fig. 1b at the expense of generating low spatial frequency interferograms (Fig. 1d–e,g–h) and FT method fails to recover lossless phase information of the specimen. Alternative option is to implement temporal phase shifting (TPS) based phase recovery algorithms^13,14^ while using low TC light sources for full FoV phase reconstruction of the specimens without sacrificing spatial resolution^5,7,15^. The compromise with this route is reduced temporal resolution as TPS requires multiple phase shifted interferograms for the phase recovery of the specimens^15^. Consequently, low TC light source reduces the space–time bandwidth product of QPM, which is not ideal for highly dynamic biological studies.

Recently, to address these issues in QPM systems, spatially low and temporally coherent light source also called pseudo-thermal light source (PTLS), have been employed in QPM^3,6,9,16–20^. PTLS is generated when a highly coherent laser beam is passed through a rotating diffuser, the output of the diffuser has high temporal and low spatial coherence properties^19^. This enables high space–time bandwidth product with high spatial phase sensitivity in QPM^3^. Despite its conspicuous advantages, there are still some limitations of PTLS, namely: spatial phase sensitivity is poorer than low TC illumination^3^, rotating diffuser could introduce vibration in QPM if the system is unstable, the light source is originated from laser and is not similar to WL which is most widely used light source in the optical microscopes due to its bio-compatible nature. Recently, there is a growing interest to apply QPM for in-vitro fertilization (IVF) for detecting sperm abnormalities^2,4,21^. Here, the use of WL in QPM could alleviate the problems associated with using coherent light sources like laser for its utilization in the medical field.

Here we utilize spectrally filtered WL based QPM in combination with single-shot high resolution Hilbert Spiral Transform (HST) based phase recovery algorithm^22^. The WL source was spectrally filtered using a narrow bandpass filter of 10 nm bandwidth (Thorlabs # FL632.8–10) to improve its TC properties generating moderately high fringe density in Linnik based QPM. The proposed route enabled the use of HST algorithm for high resolution phase recovery of the specimens over the entire FoV supported by the camera. Contrary, it was demonstrated that FT method with similar fringe density cannot reconstruct the phase of the specimens with full spatial resolution. The high spatio-temporal bandwidth of single-shot HST based LC-QPM was demonstrated first on static samples such as nano-fabricated structures and fixed Mouse Embryonic Fibroblasts (MEFs) and then for high-speed imaging of human red blood cells (RBCs) and sub-cellular structures in living human osteosarcoma U2OS cell.

To exhibit the potential of the HST method, we have conducted extensive simulations with a range of fringe patterns, including straight, curved, and circular fringes. The simulations are provided in the supplementary materials, and they proved that HST algorithm supports both the highly curved and the circular fringes, where FT method fails to recover the phase. Taking advantage of this feature, we demonstrated that HST based LC-QPM supports scalable FoV by enabling user-defined imaging objective lenses of different magnifications. Different objective lenses 10 × /0.25NA, 20 × /0.45NA, 60 × /0.9NA, and 60 × /1.2NA are utilized in the object arm while keeping 10 × /0.25NA objective lens in the reference arm of QPM. Thus, objective lens switching in the object arm required the adjustment of OPD to form interference fringes in low coherence QPM. OPD adjustment generated either curved or circular fringes for different objective lenses in the object arm due to the wavefront curvature mismatch in both arms of QPM. The largest OPD adjustment was required for 60 × /1.2NA objective lenses and introduced largest curvature mismatch between both arms of QPM. Therefore, it generated highest spatial frequency circular fringes compared to other objective lenses. It was observed that FT method could not reconstruct the phase map for 60 × /0.9NA, and 60 × /1.2NA over the entire FoV. Whereas single-shot HST method^22^ was able to reconstruct full FoV phase maps with spatial resolution slightly inferior to the TPS method that required multiple frames reducing the temporal resolution. Thus, we believe that the present approach will open new avenue in the domain of high speed and highly sensitive QPM applications and paves the path of its wider penetration in future.

## Results and discussion

### Simulation study: high coherence versus low coherence

A simulation study is presented to exhibit different experimental conditions in QPM like light sources as a function of spectral bandwidth and fringe density. Figure 1 illustrates the comparison of different light sources such as laser, WL, and filtered white light (FWL) in terms of the extent of the interference FoV and fringe density. The peak wavelength of the light source is considered at 550 nm. The bandwidths of the light source are assumed to be equal to 0.001 nm (laser), 100 nm, and 40 nm (WL and FWL). For laser, only high fringe density interferogram is exhibited corresponding to the angle ${\theta }_{1}$ between two plane waves. With high fringe density interferograms, all phase reconstruction methods such as TPS, HST, and FT can be implemented without sacrificing spatial resolution and FoV. On the contrary, with WL and FWL, either high fringe density interferograms at the cost of reduced FoV or low fringe density interferogram at the cost of reduced temporal resolution can be obtained. Thus, the lossless phase reconstruction can be performed either by employing FT method (for high fringe density only) or TPS method (for both low and high fringe density)^13,14^. To bridge the gap between the TPS and FT methods, we utilized HST method for the phase reconstruction using a low coherence interferogram^22^. The second and third rows in Fig. 1 illustrate the interferograms for spectral bandwidths 100 nm and 40 nm corresponding to the superposition of two plane waves at angles ${\theta }_{1}$, ${\theta }_{2}$, and ${\theta }_{3}$. It is demonstrated that all reconstruction methods can be implemented for angle ${\theta }_{1}$ (high fringe density) at the expense of reduced FoV. On the contrary, with low fringe density interferograms at angle ${\theta }_{3}$ only TPS method can be implemented for loss less phase recovery over large FoV. At angle ${\theta }_{2}$ (i.e., moderate fringe density), both TPS and HST method can be implemented for the phase recovery of the specimen. The advantage of HST over TPS is its single shot nature, which is suitable for highly dynamic live cell studies. In the present simulation study, HST method can be utilized for full FoV phase recovery at angle ${\theta }_{2}$ (Fig. 1) considering the light source spectral bandwidth of 40 nm.

### Flow chart of interferogram processing steps

It is important to note that HST phase retrieval requires high quality pre-processed interferograms^22^. The details of HST algorithm are given in the Material and Methods section. There are few main requirements: (1) sinusoidal fringe signal should oscillate around zero mean value, which means that image background should be entirely removed. (2) there should not be amplitude modulation or at least it should be slowly varying. (3) noise removal is desirable. To fulfill these requirements, image pre-processing must be performed.

**Figure 2:**
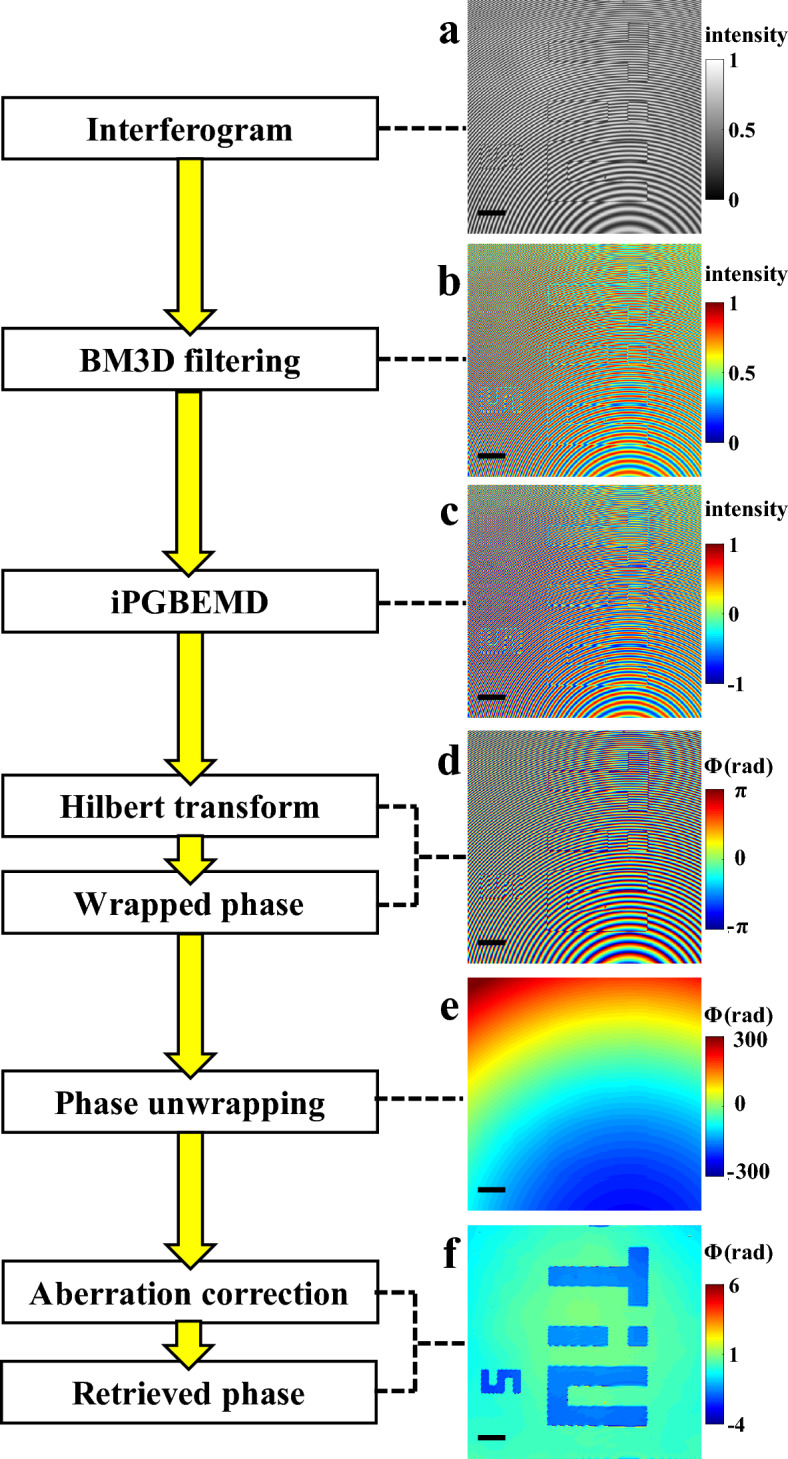
Flow chart showing main stages of phase reconstruction using iPGBEMD and HST approach: input interferogram (**a**), image after BM3D denoising (**b**), image after iPGBEMD preprocessing (**c**), wrapped phase retrieved by HST (**d**), phase unwrapped with Miguel 2D algorithm (**e**) and final reconstructed phase map after aberration correction (**f**). The scale bars are 20 μm and shown in black color solid line.

The entire phase reconstruction path is shown on a flow-chart in Fig. 2. Raw interferogram (Fig. 2a) is initially filtered by noise-removing BM3D method (Fig. 2b). Then, image is filtered by iPGBEMD^23,24^ algorithm and as a result we get fringe component within single intrinsic mode function (IMF) (Fig. 2c). iPGBEMD is a bidimensional, automatic, robust and fast version of empirical mode decomposition (EMD) method, which is further described in Section "Empirical mode decomposition". iPGBEMD is a data driven method, because image decomposing does not require any input parameters and it is based on the extrema distribution within the signal. After iPGBEMD fringe component is detached from any background and it oscillates around 0 mean value. Subsequently, HST^22^ is performed on this IMF and as a result wrapped phase map is received (Fig. 2d). The next step is the phase unwrapping performed by Miguel 2D Unwrapper algorithm (Fig. 2e)^25^. At the end, linear and spherical phase components are removed from the phase by employing principal component analysis (PCA) algorithm^33^; the final phase map is shown at Fig. 2f.

### Comparison of different phase reconstruction algorithms on a test object

First, we systematically compared different phase reconstruction algorithms such as TPS, FT and HST in terms of the spatial resolution of the recovered phase maps. To conduct this study, a standard step object of UiT text (Height = 110 nm) is placed under QPM system (Fig. 8) to record interferometric images. The objective lens 20 × /0.45NA is used in the object arm keeping 10 × /0.25NA objective lens in the reference arm of QPM. Since the optical path lengths of 10 × /0.25NA and 20 × /0.45NA objective lenses are not equal, therefore, the unit of reference arm objective lens and mirror is translated by 5.08 mm to observe interference fringes in QPM.

**Figure 3:**
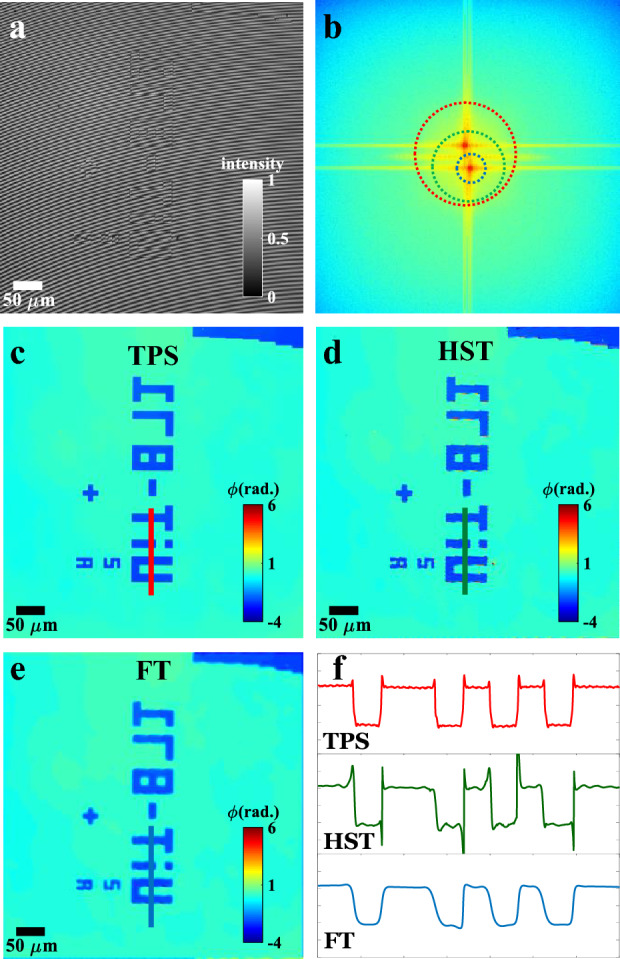
Reconstruction of the phase map originating from structures fabricated by etching  silicon wafer, objective lens 20 × /0.45NA. (**a**) Input interferogram, (**b**) Fourier spectrum with 3 circles showing: extent of the zero-order peak (red), part of the spectrum related to carrier spatial fringes (green) and part of the spectrum selected for phase reconstruction to avoid overlapping (blue). (**c**–**e**) Comparison of phase reconstruction by TPS, HST, and FT, respectively. (**f**) Line profiles of part of the phase marked with blue, green and red lines on the phase maps.

To compare the performance of TPS, FT and HST algorithms, multiple phase shifted interferograms of step object (UiT text) are recorded. Multiple phase shifted frames are used in PCA based TPS method for the phase retrieval^13,14^. However, only one of the interferograms is used in FT and HST method for single-shot phase recovery. The interferogram of UiT text is depicted in Fig. 3a. Figure 3b illustrates the Fourier spectrum of the interferogram. It can be clearly visualized that zero, + 1 and -1 orders have significant spectral overlap in the Fourier spectrum and thus makes it difficult to recover lossless phase recovery of the specimen using FT method. The extent of spectral bandwidth of the zero-order peak is shown in red dotted circle, blue dotted circle represents spectral components of + 1 order peak without spectral overlap with 0 and -1 order peak. Green dotted circle shows the extent of + 1 order spectral component, which is required to be filtered out for lossless phase reconstruction of the specimen. Thus, it is evident that in FT method, there is a loss of resolution when a small window is opted to remove the spectral overlap.

Figure 3c–e exhibit recovered phase maps corresponding to TPS, HST, and FT methods, respectively. The line profiles along the UiT text marked with red, green, and blue color solid lines in the recovered phase maps are illustrated in Fig. 3f. TPS method provided the highest possible spatial resolution in the recovered phase map at the expense of reduced temporal resolution. The single-shot FT method required only single interferogram for the phase reconstruction, however, it failed to recover the phase map with high spatial resolution. The edges of the structures in FT recovered phase map are not sharp like TPS method, thus loss in the spatial resolution. This is due to the significant overlap in the Fourier peaks, which made it impossible to recover the phase map with full spatial resolution using FT method. On the contrary, HST method allowed us to recover the phase map with moderately high spatial resolution from single interferogram.

### Scalable FoV low coherence phase imaging: test object

In this section, different objective lenses 10 × /0.25NA, 20 × /0.45NA, and 60 × /0.9NA, are sequentially utilized in the object arm of low coherence QPM keeping fixed 10 × /0.25NA objective lens in the reference arm to demonstrate scalable FOV and resolution. Different objective lenses required OPD adjustment between the object and the reference arm of QPM system to form interference fringes with low TC light source. First, identical objective lenses 10 × /0.25NA are used in both the arms of QPM to know the zero OPD position of the object and the reference arm of QPM system. This position is noted as a reference position. Next, the objective lenses 20 × /0.45NA and 60 × /0.9NA are sequentially inserted only in the object arm and corresponding zero OPD positions are found to be 5,08 mm and 15,16 mm, respectively, from the reference position.

**Figure 4:**
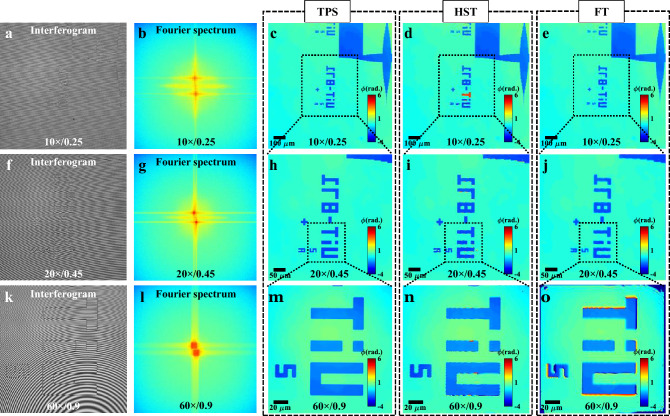
Scalable FOV and resolution phase imaging. (**a**, **f**, **k**) Low coherence interferograms acquired with 10 × /0.25NA, 20 × /0.45NA, and 60 × /0.9NA objective lenses, respectively. (**b**, **g**, **l**) Corresponding Fourier spectrums. (**c**–**e**, **h**–**j**, **m**–**o**) Reconstructed phase of the test object (H = 110 nm) corresponding to 10 × /0.25NA, 20 × /0.45NA, and 60 × /0.9NA objective lenses, respectively, by employing TPS, HST, and FT methods.

Figure 4a,f,k represent low coherence interferograms of the test object for 10 × /0.25NA, 20 × /0.45NA, and 60 × /0.9NA objective lenses, respectively. The Fourier spectrums of the interferograms are illustrated in Fig. 4b,g,l. The Fourier peaks have significant spectral overlap in the Fourier domain and introduce difficulty in phase recovery of the specimen with full spatial resolution using FT method. The ground truth high resolution phase images of the test object are generated using TPS^13,14^ and illustrated in Fig. 4c,h,m corresponding to 10 × /0.25NA, 20 × /0.45NA, and 60 × /0.9NA objective lenses, respectively. The phase reconstructions using single-shot HST, and FT methods are exhibited in Fig. 4d,i,n and e,j,o, respectively. HST method can reconstruct the phase maps with high spatial resolution compared to FT method. FT method reconstructed phase maps of the structures are smoother at the edges suggesting spatial resolution loss. Thus, HST method allowed high spatio-temporal bandwidth product in low coherence QPM. It is demonstrated that scalable FOV and resolution phase imaging with high temporal resolution is possible using low coherent light sources.

### Scalable FOV low coherence phase imaging: MEF cells

**Figure 5:**
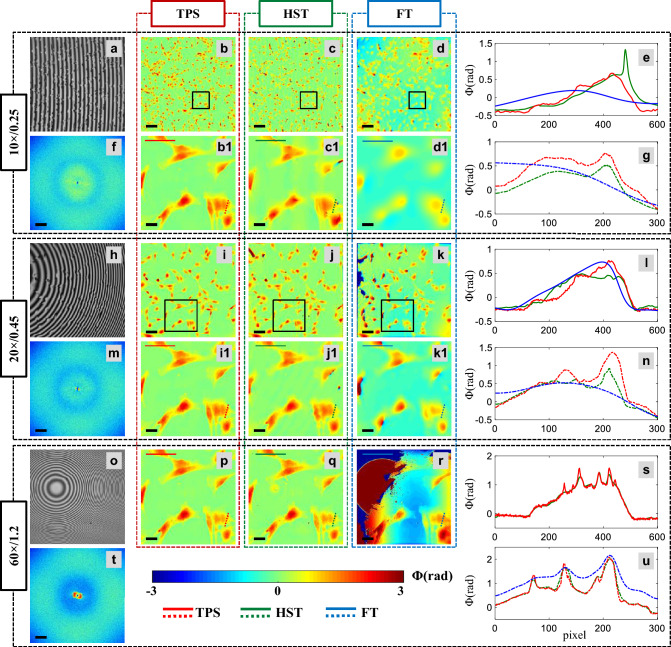
Scalable FOV and resolution low coherence phase imaging. (**a**, **h**, **o**) Low coherence interferograms of MEF cells recorded with 10 × /0.25NA, 20 × /0.45NA, and 60 × /1.2NA objective lenses. (**f**, **m**, **t**) Fourier spectrums. (**b**–**d**, **i**–**k**, **p**–**r**) Corresponding recovered phase maps of MEF cells for TPS, HST, and FT methods, respectively. (b1–d1, i1–k1) Zoomed views of the regions marked with black solid boxes in full FOV phase images for 10 × /0.25NA, and 20 × /0.45NA objective lenses. Zoomed views correspond to full FOV for 60 × /1.2NA. (**e**, **g**, **l**, **n**, **s**, **u**) The line profiles of the MEF cell’s phase maps along red, green and blue color solid and dotted lines. The scale bars are 100 μm, and 50 μm for 10 × /0.25NA, and 20 × /0.45NA objective lenses, and 20 μm for 60 × /1.2NA and zoomed views. Scale bars are shown in black color solid line.

To demonstrate the potential of the present approach in real bio application, experiments are conducted on fixed Mouse Embryonic Fibroblast (MEF) cells. MEF cells are prepared on a reflecting substrate (Si-wafer) and placed under low coherence QPM system for interferometric recording. Figure 5a,h,o depict low coherence interferograms of MEF cells recorded with 10 × /0.25NA, 20 × /0.45NA, and 60 × /1.2NA objective lenses, respectively. The corresponding Fourier spectrums are exhibited in Fig. 5f,m,t. The reconstructed phase maps obtained from TPS, HST, and FT methods for 10 × /0.25NA, 20 × /0.45NA, and 60 × /1.2NA objective lenses are illustrated in Fig. 5b–d,i–k and p–r, respectively. The corresponding zoomed views of the regions marked with black solid boxes are presented in Fig. 55b1–d1 and i1–k1, respectively to clearly demonstrate the difference in spatial resolution obtained from TPS, HST, and FT methods. The line profiles of the zoomed phase maps along the red, green, and blue solid and dotted lines for 10 × /0.25NA, 20 × /0.45NA, and 60 × /1.2NA objective lenses are depicted in Fig. 5e,g,l,n,s,u, respectively, corresponding to TPS, HST and FT methods. Although the spatial phase resolution of HST method is found to be little worse than TPS method, the advantage of HST over TPS is high temporal resolution which enables high speed phase imaging of dynamic samples as performed in the next section. It can be visualized that the reconstructed phase maps obtained from HST method preserve high spatial frequency details of the MEF cells compared to FT method. For 10 × /0.25NA and 20 × /0.45NA objective lenses FT reconstruction delivers very poor resolution, while for 60 × /1.2NA FT reconstruction is failed due to the highly curved interference fringes. Thus, HST reconstruction method supports user-defined imaging objective lens, i.e., scalable FoV in unbalanced QPM set-up which are prone to highly curved interference fringes.

### High resolution live cells imaging

The proposed approach is next applied to both long-term and real-time imaging of a living cells. Long time-lapse of living U2OS cells was recorded with 60 × /1.2NA objective lens. U2OS cells were prepared on reflecting Si-wafer. The interferometric movie of the sample was recorded for total of almost 3 h with an interval of 30 s. Phase was retrieved from raw interferograms by using HST-based procedure^22^, which is shown in Fig. 2. Time-lapse was made using ImageJ software.

**Figure 6:**
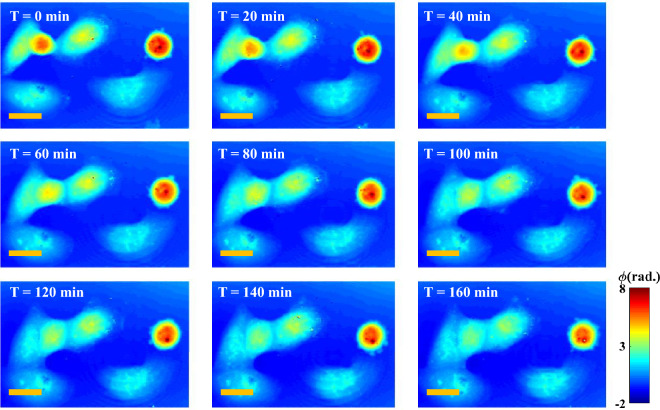
Time-lapse reconstructed phase maps of U2OS cells for 3 h recorded with 60 × /1.2NA objective lens. Associated entire time-lapse movie is given in Supplementary Video S1. The scale bars are 20 μm and shown in yellow color solid lines.

The results show that method is fully applicable to live cell imaging—despite of the presence of some artifacts, the phase reconstruction quality in terms of spatial resolution is good, and activity of subcellular structures can be observed. As the adopted phase reconstruction method (i.e., HST) is single shot, therefore, we believe that the present approach can be used to analyze fast moving samples (like live sperm cells, nanoscopic objects etc.) with an acquisition speed limited by the camera. The reconstructed phase maps of U2OS cells at different time points are illustrated in Fig. 6. Entire time-lapse interferometric and phase movie can be found in Supplementary Video S1 and S2. Harnessing the high spatial sensitivity of low coherence QPM and  single-shot phase recovery using HST method^22^, sub-cellular dynamic of organelles inside the U2OS cells were monitored over long period of intervals. In summary, the label-free nature, coupled with the application of white light, forms a highly conducive approach for live cell studies, enabling observation of specimen function over extended durations.

**Figure 7:**
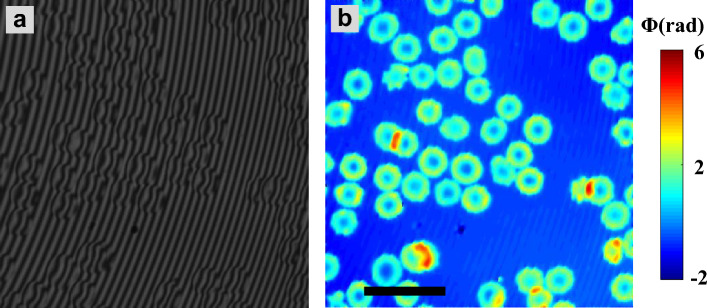
Interferogram (**a**) and reconstructed phase map (**b**) of live RBCs recorded with 20 × /0.45NA objective lens. Associated entire time-lapse movie is given in supplementary video S3. The scale bar is 15 μm and shown in black color solid line.

Further, the high-speed imaging capability of the present method is demonstrated by real time imaging of human RBCs. Live RBCs were imaged for total of 6 s at a rate of 50 frames per second. The reconstructed phase map is illustrated in Fig. 7. Entire phase movie can be found in Supplementary Video S3. Despite the artifacts caused by abrupt phase jump on the edges of the cells, overall quality of the phase map is satisfying. These results prove that presented approach is suitable for high-speed live cell imaging, where acquisition speed of the camera is the only limit of the throughput.

## Conclusion

Low TC light sources bring various advantages such as speckle noise free and coherent noise free phase imaging of the specimens with high spatial resolution and high spatial sensitivity. However, QPM systems implemented with low TC light sources allow quantitative phase imaging of the biological specimens at low temporal resolution due to the requirement of multiple phase shifted frames for phase recovery. Thus, current low coherence QPM solutions provide reduced spatio-temporal bandwidth product. In the present work, we aim to provide a hybrid, experimental and numerical approach to provide high spatio-temporal bandwidth product in low coherence QPM. A systematic comparison of different phase recovery algorithms (TPS, HST, and FT) is done in terms of the spatial resolution of the recovered phase maps of the test specimen (UiT text step object; H = 110 nm). With FT method which requires high density interference fringes for lossless phase recovery, there is no way of reconstructing phase maps with high spatial resolution without sacrificing FOV. This is due to the short TC length of the light source utilized in low coherence QPM. Short TC of the light source either forms high-density interference fringes in a limited FOV or low-density fringes over the entire FOV.

Compared to TPS method, HST is a single-shot high resolution phase recovery method and does not require very high fringe density for loss less phase recovery like FT method. Thus, the throughput of the low coherence QPM system can be increased by employing HST method for phase recovery with minor loss in spatial resolution. Observations indicate that while the HST method produces images with moderate resolution, exceeding that of the Fourier transform (FT) method, it may fall short of achieving resolution levels comparable to those attained with TPS. Additionally, HST tends to generate minor artifacts along the edges of objects exhibiting sharp discontinuities.

With the proposed approach, scalable FOV and resolution phase imaging of the test specimen and a biological specimen (MEF cells) is demonstrated. Furthermore, this approach is utilized to capture the sub-cellular dynamics within U2OS cells for an extended duration of 3 h, as well as to observe the high-speed dynamics of human RBCs at a rate of 50 fps. We believe the proposed approach of achieving high throughput phase imaging with high spatial resolution and high phase sensitivity will open new avenues of bioimaging where both high-spatial sensitivity and high-imaging speed is necessary, such as live cell imaging of sperm cells^3^, pathogens like bacteria^26^ and nanosized bio-particles such as liposomes^27^, extra-cellular vesicles and exosomes.

## Materials and methods

### Empirical mode decomposition

Empirical Mode Decomposition (EMD) is one of the most interesting approaches for fringe pattern pre-processing. Norden Huang invented and described EMD in 1996^28^ as a method for nonlinear and nonstationary data analysis. EMD is investigating extrema distribution within the signal and through analysis of this distribution it decomposes the signal into so-called intrinsic mode functions that represent different scales/frequencies in the signal. Initial version of EMD algorithm was working only in one dimension, but later its bidimensional version has been developed (BEMD)^29,30^, which allowed for preprocessing interferograms with it, in example detaching fringe component from noise and background^31,32^. As BEMD was not perfect and highly time-consuming, several improvements have been proposed over the years, like: Fast Adaptive Bidimensional EMD (FABEMD)^33^, Enhanced Fast EMD (EFEMD)^34^ and Period-Guided BEMD (PGBEMD)^35^. In the present work, we used one of the newest developments in the field—improved PGBEMD (iPGBEMD)^23^ that offers improved computation time and adaptive window size for extrema determination (which prevents so-called mode mixing phenomenon—spreading of the information among several modes or mixing inside one^36^). In this application iPGBEMD is used to improve the interferogram quality and thus make phase retrieval easier for HST.

### Hilbert spiral transform (HST): phase recovery algorithm

We utilized Hilbert Spiral Transform (HST) for the phase reconstruction^37^. HST is a single-shot technique, which can be utilized to investigate rapidly moving biological objects. It is a clear advantage over multi-frame approaches like TPS^13,14^. Moreover, HST has two main advantages over the most popular single-shot method—Fourier Transform. First, sometime FT needs a manual window selection in spectral domain, which makes automatization difficult especially with the highly curved and spherical fringes. Recently, convolution neural network has been implemented for the automated adaptive window selection in the Fourier domain^38^. This is not the case in HST approach. Second, HST is far less dependent on fringe density and shape. FT requires very dense fringes to reconstruct the phase properly. It fails to reconstruct the phase map over entire FOV with highly curved/circular fringes as demonstrated in the later sections. Such highly curved/circular fringes are inherent in low coherence QPM system when high resolution and high magnification objective lens is used only in the object arm of the system.

HST carries a unique feature of numerical generation of second phase-shifted frame from the recorded interferogram. The phase retrieval uses an analytic signal s_A_, which can be expressed as^39^:

1 $${s}_{A}\left(x,y\right)=s\left(x,y\right)+i{s}_{H}\left(x,y\right),$$

where its real part *s(x,y)* is the input interferogram and imaginary part *s*_*H*_*(x,y)* represents phase shifted interferogram obtained from HST and can be expressed follows^22^:

2 $${s}_{H}=-iexp\left(-i\beta \right){F}^{-1}\left\{P\left({\zeta }_{1},{\zeta }_{2}\right)F\left[s\left(x,y\right)\right]\right\},$$

where β is local fringe orientation map, F stands for Fourier transform, F^−1^ represents inverse Fourier transform, $P\left({\zeta }_{1},{\zeta }_{2}\right)$ is the spiral phase function and (ζ1, ζ2) are the frequency domain coordinates.

Here, 2D extension of 1D Hilbert transform called Hilbert Spiral Transform is utilized for the generation of phase shifted interferogram from the original one^22,37,40^. HST uses pure spiral phase function as a 2D spiral extension of the signum function, which allows to maintain circular symmetry, achieve isotropy and avoid the problem of upper half-plane analysis. The spiral phase function in frequency domain can be expressed by the following relation:

3 $$P\left({\zeta }_{1},{\zeta }_{2}\right)=\frac{{\zeta }_{1}+i{\zeta }_{2}}{\sqrt{{\zeta }_{1}^{2}+{\zeta }_{2}^{2}}}$$

The wrapped phase map of the specimens can be measured by employing the following relation

4 $$\phi \left(x,y\right)={{\text{tan}}}^{-1}\left[\frac{{s}_{H}\left(x,y\right)}{s\left(x,y\right)}\right]$$

The phase unwrapping is performed by using Miguel 2D phase unwrapping algorithm, which removed the phase ambiguities and provide the actual phase map of the specimen. All experimental results were acquired by CMOS camera with image size of 2304 × 2304 pixels. Computing time of the algorithm (all phase reconstruction steps combined) was around 5 min for medium-advanced personal computer with the following specifications: Lenovo Legion, CPU: Intel(R) Core(TM) i7-9750HF @ 2.60 GHz, GPU: NVIDIA GeForce RTX 2060, 16 GB RAM memory.

### Sample preparation

The cells were split and cultured in Dulbecco's modified Eagle's medium (DMEM) - high glucose with added 10% fetal brovine serum (FBS) and 1% Penciline-streptomycin. The cells were kept in a humidified 5% CO2 incubator in standard culture conditions at 37 °C. The cells were divided when they reached 80- 90% confluency, approximately every 3–5 days. For the imaging, cells were seeded in a PDMS chamber on reflecting Si substrate for 24 h in the incubator. For live cell imaging, a coverslip was kept directly on the top of PDMS chamber containing the live cells and imaging was carried out within 3 h at the room temperature. For fixed cell imaging, cells were first fixed with 4% PFA fixative. Further, the cells were washed 3 times for 5 min to remove extra PFA and imaging was performed by following the similar protocol for the live cell imaging. The individual required ingredients were bought from Thermo Fisher Scientific. MEF cell line was purchased from American Type Culture Collection (ATCC, SCRC-1008). U2OS cell line was purchased from European Collection of Authenticated Cell Cultures (ECACC, # 92,022,711).

### Experimental setup: Linnik interferometer based QPM

**Figure 8:**
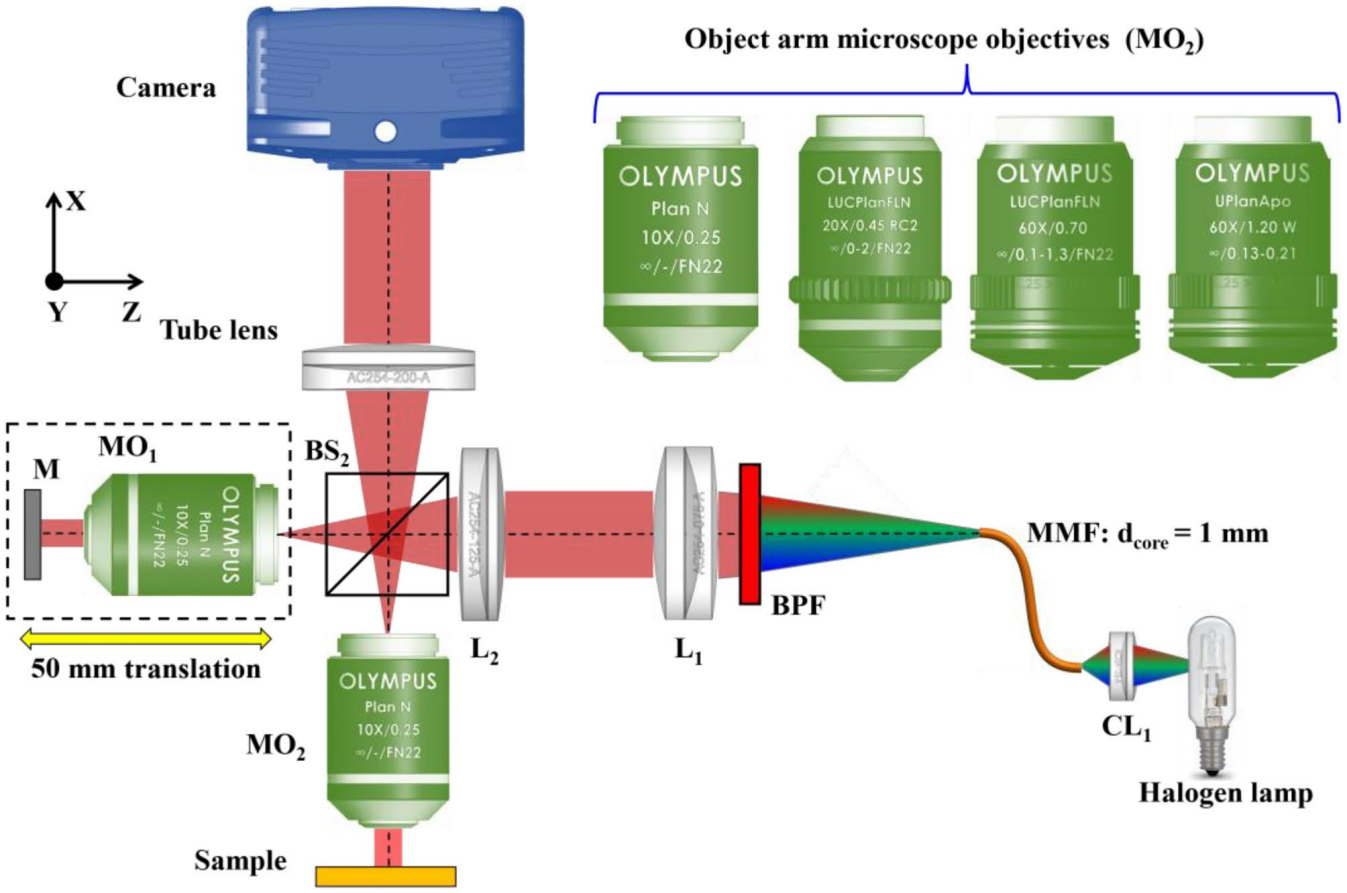
Schematic diagram of low coherence QPM. MO_1–2_: Microscope objectives; BS: Beam splitter; L_1–2_: Achromatic doublet lenses; CL: Coupling lens; BPF: Bandpass filter; MMF: Multi-mode fiber; M: Mirror.

The schematic of the experimental setup of Linnik interferometer based QPM system is illustrated in Fig. 8. The white light beam coming from a halogen lamp is coupled into a multimode fiber (MMF) of core diameter of 1 mm using lens CL_1_. The output light of MMF is filtered by inserting a narrow bandpass filter (peak wavelength: 632 nm; spectral bandwidth: 10 nm) into the light path and coupled into interference microscopy system. The output of MMF is collimated using lens L_1_ and then focused at the back focal plane of the objective lens using lens L_2_. The input light beam is split into the object and the reference beam using beam splitter BS; one is directed towards the specimen arm and the other is directed towards reference arm of QPM. The light beams coming back from the sample and the reference mirror are recombined at BS and superimposed at the camera using a tube lens to form interference pattern of the specimen. The resulting interference fringes are processed to recover the phase map of the specimen. The unit of reference arm objective lens and mirror are mounted on a translation stage of 50 mm travel range to allow the OPD adjustment between the object and the reference arm of QPM. This helps to form interference fringes in low coherence QPM when different objective lenses are changed only in the object arm keeping fixed objective lens in the reference arm.

### Supplementary Information

Supplementary Legends.

Supplementary Video S1.

Supplementary Video S2.

Supplementary Video S3.

## Data Availability

The authors declare the availability of the data used in the research to obtain the results reported in the manuscript upon reasonable request from the corresponding author.
