# Supplementary material for: High space–time bandwidth product imaging in low coherence quantitative phase microscopy
Source: Sci Rep. 2024 Apr 22;14:9191. doi: 10.1038/s41598-024-59874-y (PMC11035680; doi:10.1038/s41598-024-59874-y)
Supplement: Supplementary file 1 — Supplementary Legends. [file 41598_2024_59874_MOESM1_ESM.docx]

**Supplementary information**

**High space-time bandwidth product imaging in low coherence quantitative phase microscopy**

Azeem Ahmad^1,3,*^, Paweł Gocłowski^1,3^, Vishesh Dubey^1^, Maciej Trusiak^2^, Balpreet S. Ahluwalia^1^

^1^Department of Physics and Technology, UiT The Arctic University of Norway, 9037 Tromsø, Norway

^2^Warsaw University of Technology, Institute of Micromechanics and Photonics, 8 Sw. A. Boboli St., 02-525 Warsaw, Poland

^3^Authors contributed equally to this work.

*Corresponding author: [ahmadazeem870@gmail.com](mailto:ahmadazeem870@gmail.com)

**Supplementary simulations:** we've conducted extensive simulations with a range of fringe patterns, including straight, curved, and circular fringes (Supplementary Figs. S1, S2 and S3, respectively). By varying the spatial frequencies of these simulated interferograms, we aim to illustrate the limitations and advantages of both the FT and HST phase recovery methods. These datasets have been numerically generated using temporal phase shifting (TPS) and the experimental data of HeLa cells, providing a comprehensive understanding of the method's capabilities.


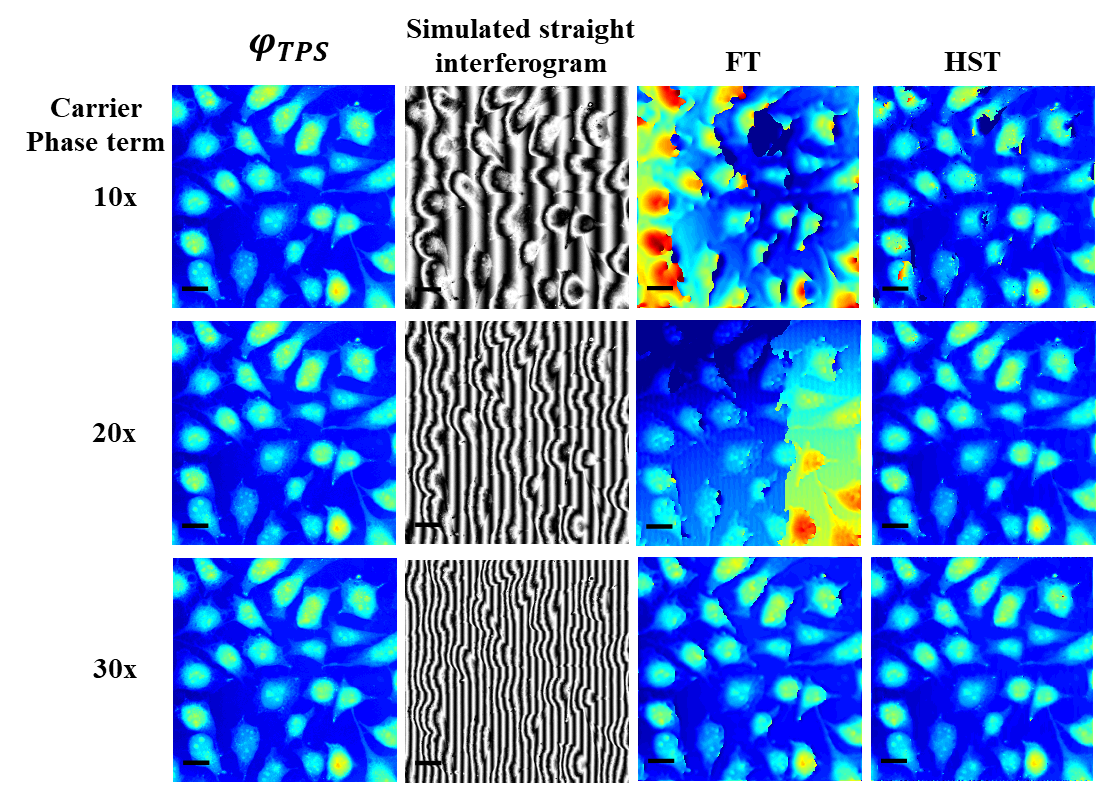


Supplementary Fig. S1. Performance comparison of HST and FT phase retrieval methods for 3 different densities of linear fringes. 1^st^ column shows ground truth phase maps obtained from TPS, and 2^nd^ column shows numerically generated fringe patterns with 10, 20, and 30 fringes over entire FOV. 3^rd^ and 4^th^ column show phase maps retrieved from simulated interferograms with FT and HST, respectively. HST reconstruction has significant advantage over FT for lower densities of linear fringes.


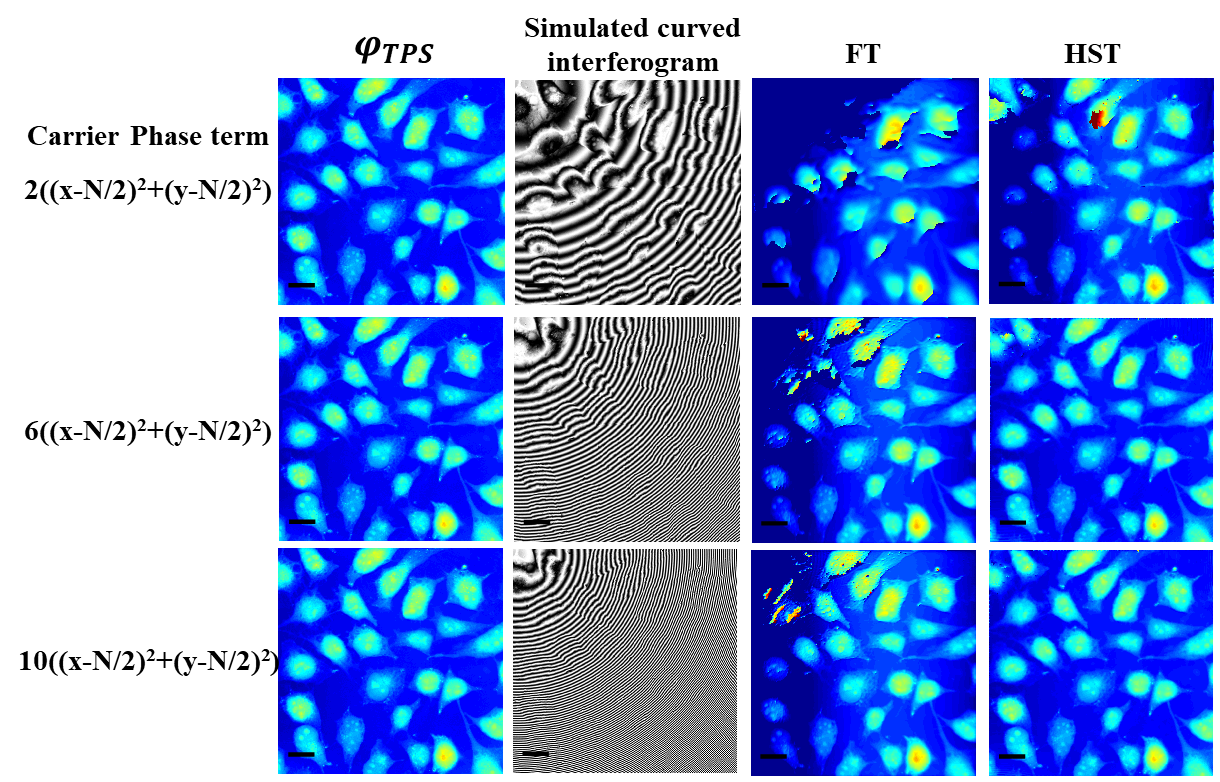


Supplementary Fig. S2. Performance comparison of HST and FT phase retrieval methods for 3 different densities of curved fringes. 1^st^ column shows ground truth phase maps, retrieved from experimental data by TPS method. 2^nd^ column shows fringe patterns, which were numerically generated in MATLAB by using equations like: *interferogram = cos (*$\varphi_{TPS}$ *+*$\varphi_{career}$*)* etc. 3^rd^ and 4^th^ column show phase maps retrieved from simulated interferograms with FT and HST, respectively. HST reconstruction has a clear advantage over FT for higher densities of curved fringes. The career phase term ‘$\varphi_{career}$’ is in the form of ((x-N/2)^2^+((y-N/2)^2^), where N is the number of pixels.


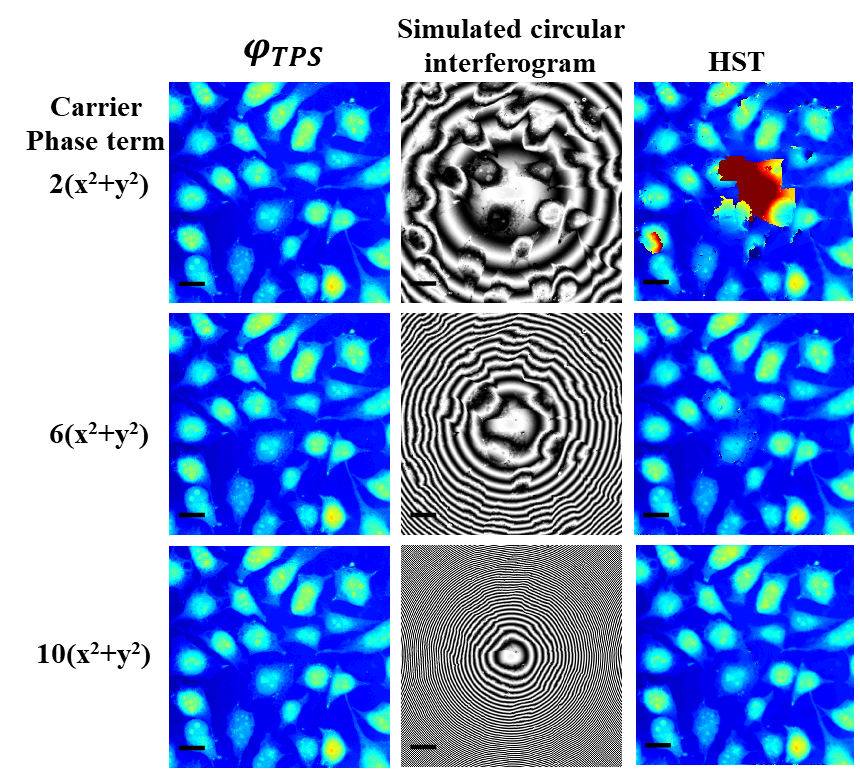


Supplementary Fig. S3. Performance of HST phase retrieval method for 3 different densities of spherical fringes. 1^st^ column shows ground truth phase maps, and 2^nd^ column shows numerically generated fringe patterns. 3^rd^ column shows phase maps retrieved from simulated interferograms with HST. FT reconstruction is not included here, because it is not possible to perform it for closed spherical fringes. Therefore, HST has a clear advantage over FT regardless of fringe density. HST, however, is not recommended to use for very sparse spherical fringes.

**Supplementary Video S1:** Time lapse interferometric movie of live U2OS cells for 3h.

**Supplementary Video S2:** Reconstructed time lapse phase movie of live U2OS cells for 3h using single-shot HST method.

**Supplementary Video S3:** High-speed time-lapse phase movie of Human RBCs acquired at a rate of 50 frames per second.
